# Tenomodulin is essential for prevention of adipocyte accumulation and fibrovascular scar formation during early tendon healing

**DOI:** 10.1038/cddis.2017.510

**Published:** 2017-10-12

**Authors:** Dasheng Lin, Paolo Alberton, Manuel Delgado Caceres, Elias Volkmer, Matthias Schieker, Denitsa Docheva

**Affiliations:** 1 Experimental Surgery and Regenerative Medicine, Department of Surgery, Ludwig-Maximilians-University (LMU), Munich, Germany; 2 Orthopaedic Center of People’s Liberation Army, Xiamen University Affiliated Southeast Hospital, Zhangzhou, China; 3 Experimental Trauma Surgery, Department of Trauma Surgery, University Regensburg Medical Centre, Regensburg, Germany; 4 Department of Hand, Plastic and Aesthetic Surgery, LMU, Munich, Germany; 5 Novartis Institutes for Biomedical Research (NIBR), Translational Medicine Musculoskeletal Disease, Basel, Switzerland

## Abstract

Tenomodulin (*Tnmd*) is the best-known mature marker for tendon and ligament lineage cells. It is important for tendon maturation, running performance and has key implications for the resident tendon stem/progenitor cells (TSPCs). However, its exact functions during the tendon repair process still remain elusive. Here, we established an Achilles tendon injury model in a *Tnmd* knockout (*Tnmd*^*−/−*^) mouse line. Detailed analyses showed not only a very different scar organization with a clearly reduced cell proliferation and expression of certain tendon-related genes, but also increased cell apoptosis, adipocyte and blood vessel accumulation in the early phase of tendon healing compared with their wild-type (WT) littermates. In addition, *Tnmd*^*−/−*^ tendon scar tissue contained augmented matrix deposition of biglycan, cartilage oligomeric matrix protein (Comp) and fibronectin, altered macrophage profile and reduced numbers of CD146-positive cells. *In vitro* analysis revealed that *Tnmd*^*−/−*^ TSPCs exhibited significantly reduced migration and proliferation potential compared with that of WT TSPCs. Furthermore, *Tnmd*^*−/−*^ TSPCs had accelerated adipogenic differentiation accompanied with significantly increased peroxisome proliferator-activated receptor gamma (*Pparγ*) and lipoprotein lipase (*Lpl*) mRNA levels. Thus, our results demonstrate that Tnmd is required for prevention of adipocyte accumulation and fibrovascular scar formation during early tendon healing.

Tendon injuries are some of the most common orthopedic problems accounting for substantial pain, disability, and economic burden.^1^ While many tendon injuries are acute, a very large number are chronic causing degenerative conditions.^2^ Repair in either case results in the formation of fibrovascular scar, fat deposition or heterotopic ossification that never attains the gross, histological, or mechanical characteristics of normal tendon.^3, 4, 5^ The precise mechanisms of matrix degeneration, tissue tearing, and the subsequent repair process remain poorly understood.^1^ Tenomodulin (Tnmd) is a member of a novel class protein family of type II transmembrane glycoproteins with a highly conserved cleavable C-terminal cysteine-rich domain.^6, 7^ The *Tnmd* gene consists of seven exons localized on the X chromosome and accounts for an ~1.4 kb transcript and a predicted full-length protein consisting of 317 amino acids.^6, 7^ It is predominantly expressed in tendons and ligaments, but low levels of mRNA transcripts have also been identified in other tissues.^6, 7, 8, 9^ *Tnmd* is the best-known marker of the mature tendon and ligament lineage with a suggested dual role of its C-terminal domain, namely a pro-proliferative action with tendon/ligament cells and anti-angiogenic potential with vascular cells.^9, 10^ Interestingly, loss of *Tnmd* expression in gene targeted mice (*Tnmd*^*−/−*^) abated tenocyte proliferation, led to reduced tenocyte density and to pathological thickening of collagen fibers in the tendon extracellular matrix (ECM) *in vivo* but caused no major changes in the tendon vasculature.^11^ In our recent study, we subjected *Tnmd*^*−/−*^ mice and their wild-type (WT) littermates to exhaustive running tests revealing significantly inferior running performance of the knockouts that further worsened with training.^12^ *In vitro* analysis of *Tnmd*^*−/−*^ tendon stem/progenitor cells (TSPCs) showed significantly reduced self-renewal, and augmented senescence paralleled by upregulated *p53* mRNA levels, which was confirmed *in vivo* by detecting an increased number of p53-positive tenocytes in *Tnmd*^*−/−*^ Achilles tendons.^13^ In addition, overexpression of *Tnmd* in murine mesenchymal stem cells (MSCs) inhibited their commitment towards the adipogenic, chondrogenic and osteogenic lineages, whilst promoting their tenogenic differentiation.^14^ The above data motivated us to further examine the potential regulatory role of *Tnmd* gene in the early tendon healing stage when major cellular and ECM events take place,^3^ such as vascular and inflammatory cell invasion, intrinsic cell activation, migration and proliferation, and ECM deposition. Hence, the objective of this study was to investigate the functions of Tnmd in early tendon healing *in vivo* and in wound healing assays *in vitro*, including careful tissue phenotyping and specific molecular target analyses, using the *Tnmd*^*−/−*^ mouse strain.

## Results

### *Tnmd*^*−/−*^ tendon scars have inferior gross appearance, histological scores and cell density paralleled with increased accumulation of adipocytes and vessels

**Figure 1:**
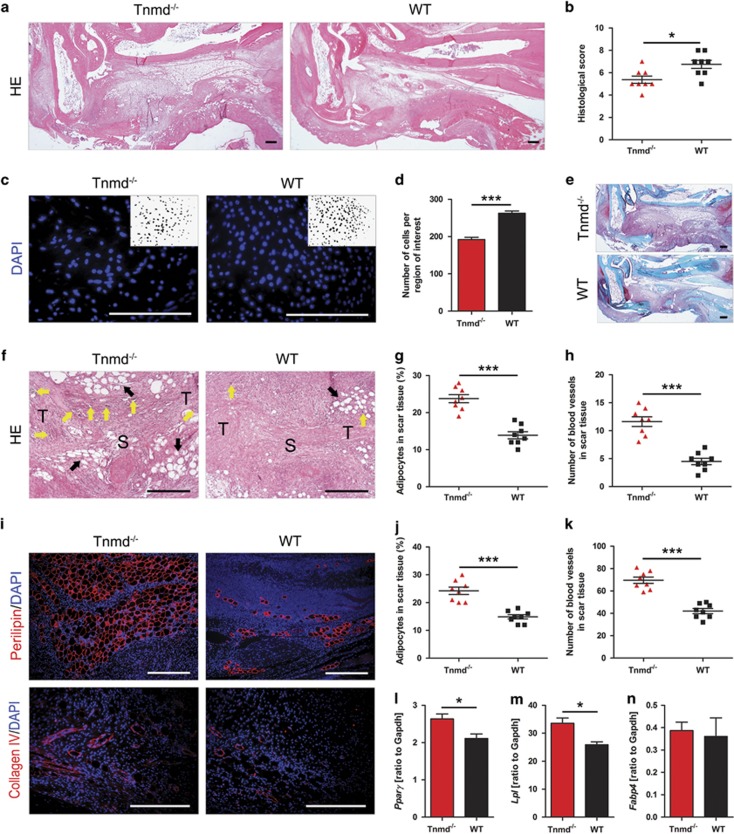
*Tnmd* deficiency results in an inferior tendon repair process, lower cell density and increased adipocyte and vessel accumulation. (**a**) Low-magnification HE staining indicates a very different scar organization with clear adipocyte accumulation in *Tnmd*^*−/−*^ mice. (**b**) Evaluation of tendon healing using an established histological scoring system revealed that *Tnmd*^*−/−*^ mice had a significantly lower total histological score at 8 days postoperatively compared with WT mice. (**c**,**d**) Cell density in the healing region was significantly lower in *Tnmd*^*−/−*^ *versus* WT mice. DAPI images were analyzed by computerized image analysis with ImageJ. (**e**) Ectopic endochondral ossification was not revealed by safranin O staining in the tendons of either genotype at day 8. (**f–h**) In HE-stained sections increased areas of adipocyte accumulation and numbers of large blood vessels were detected in the scar region of *Tnmd*^*−/−*^ tendons compared with WT mice. (**i**) Visualization of adipocytes and blood vessels in *Tnmd*^*−/−*^ and WT Achilles tendon scars via immunofluorescence staining for perilipin and collagen IV. (**j,k**) The perilipin-positive areas and number of collagen IV-labeled blood vessels were significantly higher by 8 days after surgery in *Tnmd*^*−/−*^ *versus* WT mice. (**l–n**) qRT-PCR revealed upregulated mRNA levels of *Pparγ* and *Lpl*, but no changes in *Fabp4* expression in *Tnmd*^*−/−*^ *versus* WT tendons. For quantification in (**b**, **d**, **g**, **h**, **j** and **k**), statistical significance was calculated using two-tailed non-parametric Mann–Whitney test, *n*=8 (8 animals per group; each animal represented by 3 tissue sections). For qRT-PCR in (**l**, **m** and **n**), statistical significance between 2 groups was determined by unpaired Student’s *t*-test (two-tailed) for 5 independent experiments. *P<0.05; ***P<0.001, compared with WT. S, scar; T, tendon; yellow arrows, blood vessels; black arrows, adipocytes. Scale bars: 200 *μ*m

To analyze Tnmd involvement during early tendon healing, we established a mouse model of full thickness Achilles tendon injury.^15^ We analyzed the mice eight days after surgical repair, a time point characterized by scar formation, vascular and inflammatory cell invasion, high cell migration and proliferation as well as robust ECM secretory activity.^3, 16^ Hematoxylin–eosin (HE) staining of sectioned tendons revealed a very different scar organization in *Tnmd*^*−/−*^ mice, as indicated by significantly inferior total histological scores^17^ (Supplementary Table 1) compared with their WT littermates (Figures 1a and b). Quantitatively, total cell density was significantly lower in the *Tnmd*^*−/−*^ mice at 8 postoperative days (Figures 1c and d). Ectopic ossification after tenotomy of rodent Achilles tendon at late stages of the tendon healing process has been reported in previous studies.^18, 19, 20^ However, ectopic endochondral ossification was not detected in the scar tissues in either of the genotypes following safranin O staining at 8 days post-injury (Figure 1e). In contrast, the mean area of adipocyte accumulation, the number of blood vessels observed in HE staining analyses (Figures 1f–h) and validated by immunofluorescence staining and quantification for perilipin- (Figures 1i and j) and collagen IV-positive areas (Figures 1i and k), were significantly increased in the scar sites of *Tnmd*^*−/−*^ mice compared with WT controls. We also found increased mRNA levels of the adipogenic marker genes, peroxisome proliferator-activated receptor gamma (*Pparγ*) and lipoprotein lipase (*Lpl*) in the tendons of *Tnmd*^*−/−*^ mice through quantitative reverse transcriptase PCR (qRT-PCR) (Figures 1l and m). Expression of fatty acid-binding protein 4 (*Fabp4*), another adipogenic marker, was unaffected (Figure 1n). The above data revealed for the first time that the absence of *Tnmd* leads to an inferior morphological outcome and lower cellular density, whilst it activates adipocyte accumulation and adipose-related gene expression as well as vessel numbers in the early repair region of injured tendons.

### *Tnmd*^*−/−*^ tendons demonstrate reduced cell proliferation and CD146-positive cell numbers, downregulated levels of certain tendon-related genes, whilst increasing cell apoptosis and occurrence of p53-expressing cells

**Figure 2:**
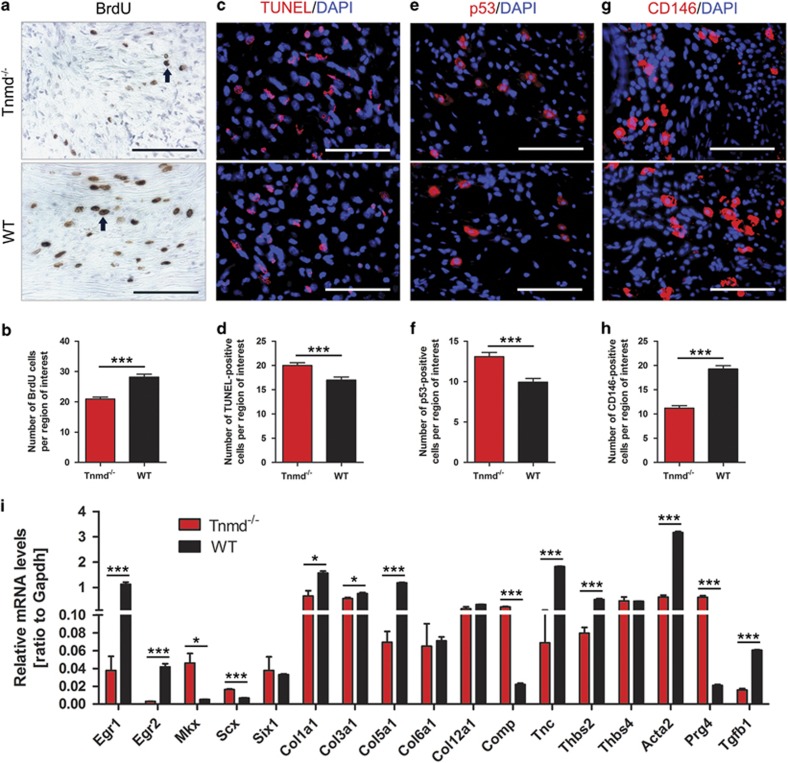
*Tnmd* deficiency results in reduced cell proliferation, CD146-positive cells, increased cell apoptosis and p53-expressing cells and an altered expression of certain tendon-related genes. (**a**,**b**) BrdU staining and quantification in the tendon scars revealed decreased proliferative cell numbers in *Tnmd*^*−/−*^ *versus* WT mice at 8 days postoperatively. (**c**,**d**) TUNEL-based analyses detecting apoptotic cells showed increased cell apoptosis in *Tnmd*^*−/−*^ mice compared with WT mice. (**e**,**f**) Increased number of p53-positive cells was found at day 8 in the tendons scar tissues of *Tnmd*^*−/−*^ tendons compared with WT mice. (**g**,**h**) Immunofluorescence staining for CD146 showed that the number of CD146-expressing cells, corresponding to local MSCs and/or TSPCs was lower in *Tnmd*^*−/−*^ mice than WT mice. (**i**) *Tnmd*^*−/−*^ tendons displayed significantly lower expression levels for *Egr1*, *Egr2*, *Col1a1*, *Col3a1*, *Col5a1*, *Tnc*, *Thbs2*, *Acta2* and *Tgfb1* compared with WT mice. In contrast, the relative gene expression of *Mkx*, *Scx*, *Comp* and *Prg4* displayed a dramatic increase. No effect was found for *Six1*, *Col6a1*, *Col12a1* and *Thbs4*. For quantification in (**b**, **d**, **f** and **h**), statistical significance was calculated using two-tailed non-parametric Mann–Whitney test, *n*=8 (8 animals per group; each animal represented by 3 tissue sections). For qRT-PCR in **i**, statistical significance between 2 groups was determined by unpaired Student’s *t*-test (two-tailed) for 5 independent experiments. *P<0.05; ***P<0.001, compared with WT. Black arrows, BrdU-positive cells. Scale bars: 100 *μ*m

To test whether the reduction in cell numbers was due to a decreased proliferation or increased apoptosis, we carried out proliferative and apoptotic assays by bromodeoxyuridine (BrdU) and terminal deoxynucleotidyl transferase-mediated dUTP-biotin nick end labeling (TUNEL) stainings. Furthermore, immunofluorescence staining was also performed for p53, which regulates the apoptosis in oxidative stress-exposed tenocytes,^21^ and has been previously shown by us to be elevated in the tendons of uninjured *Tnmd*^*−/−*^ mice.^11^ BrdU analysis confirmed a lower number of proliferating cells at the scar site of injured Achilles tendons in *Tnmd*^*−/−*^ than WT mice (Figures 2a and b). Furthermore, TUNEL assays and immunofluorescence staining for p53 showed that *Tnmd*^*−/−*^ scars had an increased number of apoptotic cells (Figures 2c–f). In order to track activated local stem/progenitor cells at the scar site, we performed immunofluorescence analysis for CD146, which labels MSCs as well as the TSPCs.^22, 23, 24^ The number of CD146-positive cells was significantly lower in *Tnmd*^*−/−*^ compared with WT mice eight days after injury (Figures 2g and h). Following this, we analyzed how the absence of *Tnmd* affects the expression levels of tendon-associated gene markers using qRT-PCR of *Tnmd*^*−/−*^ and WT tendon-derived mRNA. We observed significantly lower mRNA levels for early growth response protein 1 and 2 (*Egr1*, *Egr2*), collagens I, III and V (*Col1a1*, *Col3a1*, *Col5a1*), tenascin C (*Tnc*), thrombospondin 2 (*Thbs2*), alpha smooth muscle actin (*Acta2*) and transforming growth factor beta 1 (*Tgfb1*) in *Tnmd*^*−/−*^ samples (Figure 2i). On the contrary, the relative expression levels of mohawk (*Mkx*), scleraxis (*Scx*), cartilage oligomeric protein (*Comp*) and lubricin (*Prg4*) displayed a dramatic increase, without affecting those of sine oculis homeobox homolog 1 (*Six1*), collagens VI and XII (*Col6a1*, *Col12a1*) and thrombospondin 4 (*Thbs4*) (Figure 2i). In sum, we concluded that the loss of *Tnmd* causes simultaneously reduced numbers of BrdU- and CD146-expressing cells, but an increased incidence of TUNEL- and p53-positive cells in the tendon scar tissue and dysregulated expression of key tendon-related transcription factors and ECM genes, which in turn can lead to altered tendon tissue composition during repair.

### *Tnmd*^*−/−*^ scar tissues are characterized by erroneous ECM deposition and abnormal macrophage profile

**Figure 3:**
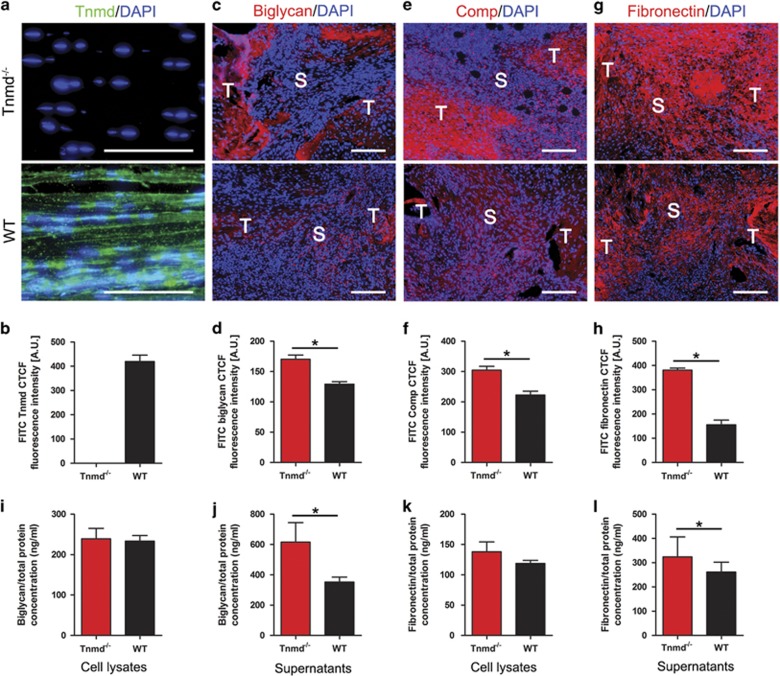
The absence of *Tnmd* increases erroneous ECM deposition. (**a**,**b**) Immunofluorescence staining with anti-Tnmd C-terminal antibody showed Tnmd secretion in the ECM of WT Achilles tendon, but not in *Tnmd*^*−/−*^. (**c–h**) Biglycan, Comp and fibronectin protein deposition in the tendon scar, analyzed by fluorescent digital signal quantification, was clearly augmented in *Tnmd*^*−/−*^ when compared with WT mice at 8 days postoperatively. (**i–l**) Biglycan and fibronectin protein levels from cell lysates and supernatant were assessed by ELISA and the levels of both proteins were significantly increased in the supernatant and slightly increased in the cell lysates of *Tnmd*^*−/−*^ *versus* WT TSPCs. For quantification in (**b**, **d**, **f** and **h**), statistical significance was calculated using two-tailed non-parametric Mann–Whitney test, *n*=3 (3 animals per group; each animal represented by 3 tissue sections). For ELISA in (**i**, **j**, **k** and **l**), statistical significance between 2 groups was determined by unpaired Student’s *t*-test (two-tailed) for two independent experiments with two donors/genotypes. *P<0.05 compared with WT. S, scar; T, tendon ends. Scale bars: 100 *μ*m

The ECM of tendon tissue is composed primarily of collagen I, as well as collagen III, elastin and various proteoglycans and mucopolysaccharides.^3^ Anomalies in the ECM composition of the scar tissue after tendon injury may contribute to a poor and delayed healing process resulting in compromised tissue quality.^20^ Prompted by this observation and the gene expression changes in *Tnmd*^*−/−*^ tendons, we carried out an ECM phenotyping of the scar tissues of both genotypes. First, we performed immunofluorescence staining with an anti-C-terminal Tnmd antibody visualizing Tnmd secretion in the ECM of WT mice Achilles tendon, but not in *Tnmd*^*−/−*^ mice (Figures 3a and b). Surprisingly, three ECM proteins, namely biglycan, Comp and fibronectin, were more expressed in *Tnmd*^*−/−*^ tendon healing sites than WT mice (Figures 3c–h). The increased protein deposition of Comp in *Tnmd*^*−/−*^ samples was consistent with the qRT-PCR data showing higher Comp mRNA levels in this group (Figure 2i). However, collagens I and III, decorin, elastin, fibromodulin and lumican were not significantly affected (Supplementary Figure 1a). Nonetheless, picrosirious red-stained tendon sections analyzed by polarized light microscopy exhibited ECM containing thicker collagen fibers in the scar areas and tendon ends of the *Tnmd*^*−/−*^ than that of WT mice (Supplementary Figure 1b). Our observation of the increased erroneous ECM deposition in the repair sites of *Tnmd*^*−/−*^ tendons motivated us to investigate by using target-specific ELISA whether *Tnmd*^*−/−*^ TSPCs secrete higher amounts of biglycan and fibronectin proteins. The obtained quantitative data further confirmed our *in vivo* results by showing that the levels of both proteins were significantly increased in the supernatant of *Tnmd*^*−/−*^ compared with WT TSPCs (Figures 3i–l).

**Figure 4:**
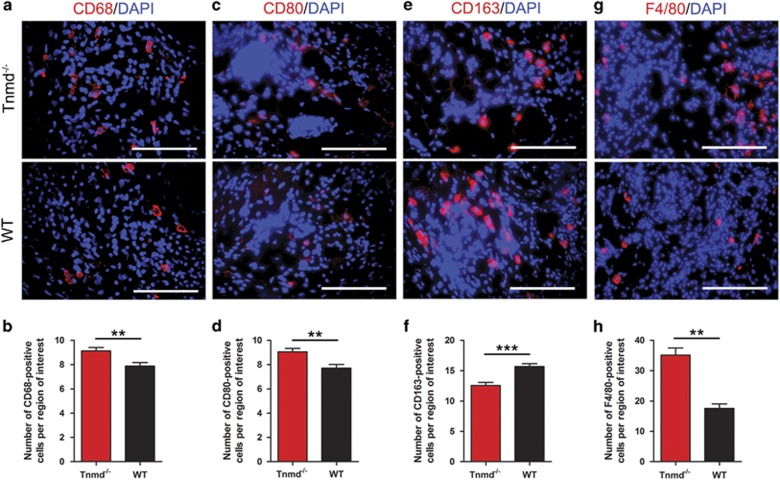
The lack of *Tnmd* alters the macrophage profile during early tendon healing. (**a–h**) The numbers of CD68-, CD80- and F4/80-positive cells were significantly increased, whereas the number of CD163-positive cells was significantly reduced in the *Tnmd*^*−/−*^ tendon scar tissues compared with WT mice. For quantification in (**b**, **d**, **f** and **h**), statistical significance was calculated using two-tailed non-parametric Mann–Whitney test, *n*=8 (8 animals per group; each animal represented by 3 tissue sections). **P<0.01; ***P<0.001, compared with WT. Scale bars: 100 *μ*m

The repair of injured tendons begins with an early inflammatory response that is associated with infiltration of pro-inflammatory, classically activated (M1) macrophages, whereas the secondary inflammatory response involves anti-inflammatory, alternatively activated (M2) macrophages.^25^ Recent evidence suggested that proper modulation of inflammation in the early stages of tendon repair may lead to improved healing.^26^ Interestingly, in our model, the numbers of cells positive for CD68, (a prominent surface marker for M1 and tissue-resident macrophages), and CD80, (a M1 macrophage surface marker), were significantly increased (Figures 4a–d), whilst the number of cells expressing CD163, (a M2 macrophage surface marker), was significantly reduced in *Tnmd*^*−/−*^ mice compared with WT mice eight days after injury (Figures 4e and f). These results were further substantiated by immunofluorescence staining of F4/80, a monoclonal antibody directed specifically against mouse macrophages, demonstrating a significant increase of labeled cells in the scar sites of *Tnmd*^*−/−*^ mice (Figures 4g and h). Collectively, this set of data shows that *Tnmd* deficiency leads to erroneous ECM deposition *in vivo* and *in vitro* and leads to an abnormal macrophage profile with pre-dominating M1 macrophages in the repair site at day 8 of tendon healing.

### *Tnmd*^*−/−*^ TSPCs show significantly lesser migratory and proliferative capacities but have accelerated adipogenic differentiation rate and significantly upregulated *Pparγ* and *Lpl* mRNA expression

**Figure 5:**
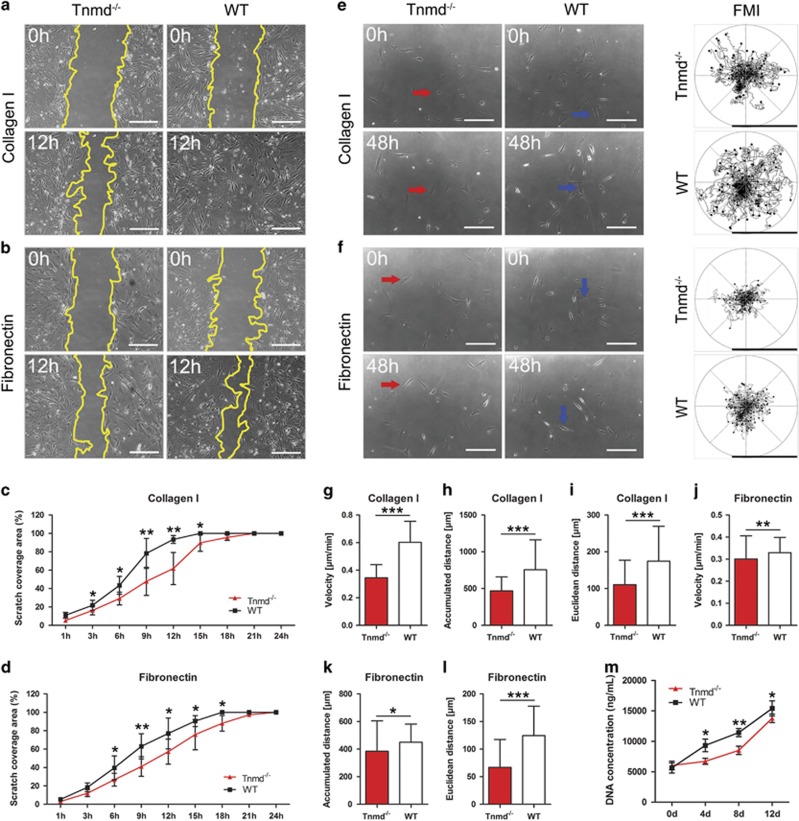
The absence of *Tnmd* in TSPCs leads to significantly reduced cell migration and proliferation. (**a**–**d**) *In vitro* wound healing assays on collagen I and fibronectin showed that *Tnmd*^*−/−*^ TSPC scratch closure was significantly slower compared with WT TSPCs. The borders of the scratches are outlined with yellow lines. (**e**,**f**) Forward migration index (FMI) plots showed that *Tnmd*^*−/−*^ TSPCs were indeed less migratory than WT TSPCs. Upper arrows on each type of matrix show the start point while lower arrows the end point of example migratory cells. (**g–l**) Quantification of velocity, accumulated and Euclidean distances further validated *Tnmd*^*−/−*^ migratory deficiency. (**m**) During 0, 4, 8 and 12 days of culture cell growth kinetics were estimated by DNA-based CyQUANT assay revealing that the proliferation of *Tnmd*^*−/−*^ TSPCs was significantly lower than that of WT TSPCs. For quantification in (**c**, **d** and **m**), *n*=4 independent experiments per group. For quantifications in random migration, *n*=3 independent experiments per group (total of 70–80 tracks per genotype). Statistical significance was calculated using two-tailed non-parametric Mann–Whitney test. *P<0.05; **P<0.01; ***P<0.001, compared with WT. Blue arrows, WT TSPCs; d, day; h, hour; Red arrows, *Tnmd*^*−/−*^ TSPCs; Scale bars: 200 *μ*m

Figures 1c and d and Figures 2a, b, g and h demonstrated that loss of *Tnmd* is associated with significantly lower cell density and numbers of BrdU- and CD146-positive cells in the scar tissues at day 8. These imply that Tnmd may regulate the migration and self-renewal capacities of TSPCs. Therefore, we carried out scratch assays mimicking wound closure *in vitro*. To estimate the effect of ECM proteins and because of the increased fibronectin deposition in *Tnmd*^*−/−*^ tendons, our scratch assays were performed on collagen I- and fibronectin-coated dishes. Quantification of scratch closure rate after 24 h, showed that the migration speed of *Tnmd*^*−/−*^ TSPCs was significantly lower compared with WT TSPCs (Figures 5a–d). This finding was further solidified by random migration analysis after 48 h, in which forward migration index (FMI) of multiple single cells migrating on either of the ECM proteins was calculated (Figures 5e and f). Quantification of velocity, accumulated and Euclidean distance, also clearly indicated a significant reduction of *Tnmd*^*−/−*^ TSPCs motility compared with WT (Figures 5g–l). Furthermore, during 12 days of culture, DNA-based CyQUANT assays at various time points showed that *Tnmd*^*−/−*^ TSPCs proliferated significantly slower than WT TSPCs (Figure 5m), confirming and expanding our earlier report that Tnmd is a positive regulator of TSPC self-renewal.^13^

**Figure 6:**
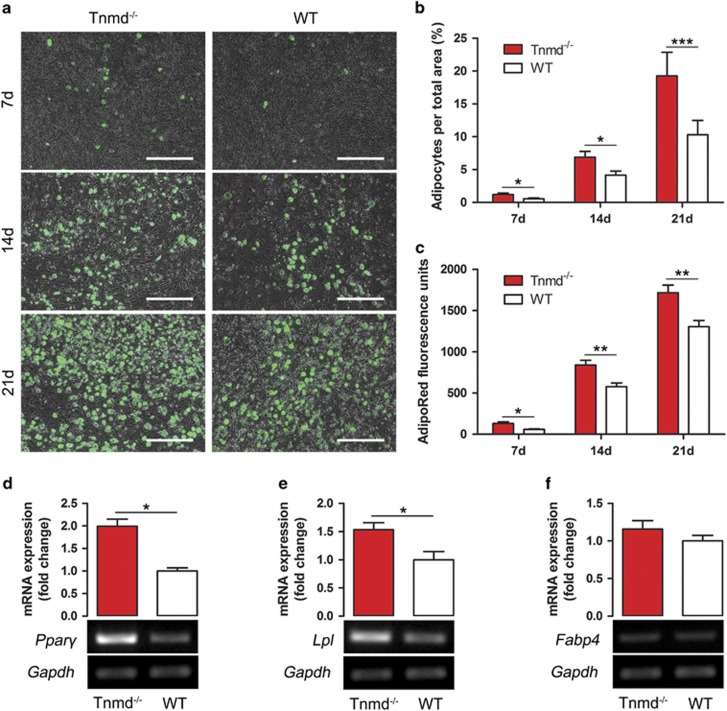
The absence of *Tnmd* in TSPCs leads to significantly accelerated adipogenic differentiation and upregulated *Pparγ* and *Lpl* expression levels. (**a**,**b**) *Tnmd*^*−/−*^ TSPCs grown in adipogenic medium had significantly more BODIPY 493/503 staining of neutral lipid droplets, indicating a higher propensity of these cells to form adipocytes than WT TSPCs after 7, 14 and 21 days, respectively. (**c**) AdipoRed fluorescent quantitative assay confirmed these results. (**d–f**) Semi-quantitative RT-PCR and densitometric band analysis revealed that the expression of the two adipogenic marker genes *Pparγ* and *Lpl* was increased, while *Fabp4* levels were not significant changed in *Tnmd*^*−/−*^ TSPCs after 21 days of adipogenic differentiation *versus* WT TSPCs. For quantification in **b** and **c** *n*=4 independent experiments. Statistical significance was calculated using two-tailed non-parametric Mann–Whitney test. For semi-quantitative RT-PCR, *n*=5 independent experiments per group. Statistical significance between 2 groups was determined by unpaired Student’s *t*-test (2-tailed). *P<0.05; **P<0.01; ***P<0.001, compared with WT. Scale bars: 200 *μ*m

The observed adipocyte accumulation during early tendon healing prompted us to test whether the loss of *Tnmd* can accelerate TSPCs differentiation into adipocytes. Previously, we have observed an increased tendency of *in vitro* adipogenesis of *Tnmd*^*−/−*^ TSPCs.^13^ Here we again subjected TSPCs to adipocyte differentiation and examined the outcome in-depth. *Tnmd*^*−/−*^ TSPCs grown in adipogenic medium had significantly more BODIPY 493/503 staining of neutral lipid droplets, indicating a higher adipogenic propensity than WT TSPCs after 7, 14 and 21 days, respectively (Figures 6a and b). Additional analysis, with the AdipoRed reagent revealed similar results (Figure 6c). Consistent with our *in vivo* results, semi-quantitative RT-PCR and densitometric PCR band evaluation showed that the expression levels of *Pparγ* and *Lpl*, but not *Fabp4*, were significantly increased in *Tnmd*^*−/−*^ TSPCs compared with those of WT following 21 days of adipogenic stimulation (Figures 6d–f). We conclude that the lack of *Tnmd* in TSPCs negatively alters their migratory and proliferative capacities, whilst accelerating their commitment towards adipocytes and the expression of critical adipose regulatory genes such as *Pparγ* and *Lpl*.

## Discussion

Effective strategies to speed up the healing process of tendon injuries are still not developed because the understanding of tendon biology lags far behind that of the other components of the musculoskeletal system, and the molecular mechanisms controlling the migration, proliferation and fate of TSPCs during tendon repair are not well understood.^1, 9^ Therefore, it is still very challenging to identify molecular targets that can be used to develop medicinal boosters for complete and timely repair of injured tendons or ligaments. *Tnmd* is a useful phenotypic marker of mature tenocytes and ligamentocytes that has been shown to have intriguing and diverse roles in developing tendons and those challenged by physical exercise.^9, 10, 11, 12, 27^ Herein, we further explored the potential roles of Tnmd in the tendon healing process by subjecting *Tnmd*^*−/−*^ mice to full thickness Achilles tendon injury and carrying out in-depth characterization of the scar tissues at day 8 as well as investigating certain TSPCs functions *in vitro*. The novel results of this study demonstrate for the first time that the absence of *Tnmd* causes inferior tendon repair process, as shown by adipocyte accumulation and fibrovascular scar formation during early tendon healing.

Efficient molecular modulation of tendon healing should accelerate cell proliferation and inhibit apoptosis, or at least not augment the number of apoptotic events.^28^ We have already reported that the tendons of *Tnmd* knockout mice exhibit reduced cell density and proliferation^11^ concomitant with apparent an *in vitro* phenotype of *Tnmd*^*−/−*^ TSPCs, which were significantly less self-renewing, and more senescent.^13^ Our current study provides further evidence that the loss of *Tnmd* expression in the healing tendon results in reduced cell density and proliferation and lower numbers of CD146-expressing cells, as well as augmented cell apoptosis and higher numbers of p53-positive cells. Furthermore, we show for the first time that *Tnmd*^*−/−*^ TSPCs also have significant migratory deficits in two different experimental set-ups. Hence, we suggest that Tnmd has anti-apoptotic and anti-senescence roles and has important regulatory roles in cell migration and proliferation during the early stage of tendon repair. The positive association of *Tnmd* expression with advancement of tendon healing was previously suggested by Tokunaga *et al.*^29^ in a growth factor-dependent model of rotator cuff healing.

Adipocyte accumulation and fibrovascular scar are common pathological changes that occur in ruptured tendons and ligaments.^30, 31, 32, 33, 34^ They often do not properly remodel and in some cases continue to worsen even after surgical repair and physiotherapy. However, little is known of the pathophysiological pathways behind these phenomena.^30, 31, 32, 33, 34^ Persistent or unresolved inflammation is considered a major trigger in many fibrotic diseases and in tendon healing has been associated with abnormal fibrogenesis.^35, 36, 37^ Recent *in vivo* large animal studies showed that inflammatory factors are dramatically upregulated within the first week after tendon injury, which in turn stimulate the production of proteases, cause apoptosis of tendon cells, impede the intrinsic repair process, and promote adhesion formation.^38, 39, 40, 41, 42^ During inflammation, macrophages have essential roles in both promoting and resolving inflammation and in both facilitating and modulating tissue repair. In an injury setting, M1 cells predominate early, whereas M2 cells accumulate later. Hence, in tendon injury, it could be postulated that M1 macrophages promote repair by stimulating ECM production and that M2 macrophages enter the process to repress inflammation and clear excess ECM, a concept that is consistent with experimental evidence.^26^ Disturbing the balance between these macrophage subtypes may result in abnormal scar formation, a defective repair process and impaired tissue function.^26^ In our model, *Tnmd*^*−/−*^ scars at day 8 exhibited higher numbers of vessels than WT scars, and displayed a macrophage profile of predominantly M1 cells with lower M2 numbers. This finding may help to explain the excess ECM protein deposition of biglycan, Comp and fibronectin, seen in *Tnmd*^*−/−*^ tendons. Similarly, some recent reports dealing with tendon healing demonstrated higher expression of biglycan and Comp in the ectopic chondro-ossification sites in injured tendon tissues.^20, 43^ In addition, Spiegelman *et al.*^44^ found that fibronectin protein can regulate adipogenic gene expression. Furthermore, a study focusing on stem cell-based therapy of tendon injuries suggested that a lower M2 macrophage number leads to less accumulation of CD146-positive cells and more erroneous matrix deposition at the repair site.^45^ Thus, we suggest that the absence of *Tnmd* leads to enhanced vascular invasion, a prolonged inflammatory response, aggravated deposition or delayed clearing of excessive erroneous ECM. A deficiency of our study is that we do not describe the precise molecular mechanism by which Tnmd regulates the above processes. At present, we are unable to elucidate the exact Tnmd mode of action because we do not yet know the binding partners of this protein. Future studies are needed if we are to decipher the Tnmd signaling pathways. It will also be important to compare our small animal model with clinical tendon pathologies, for example by investigating *Tnmd* expression levels in different tendinopathy forms.

Interestingly, many recent studies have focused on understanding *Tnmd* involvement in obesity and diabetes.^46, 47, 48, 49, 50, 51, 52^ Of interest, Senol-Cosar *et al.*^46^ suggested that *Tnmd* facilitates pre-adipocyte terminal differentiation while Jiang *et al.*^14^ showed that overexpression of *Tnmd* actually inhibits adipogenesis of murine MSCs. These data suggest that Tnmd might have cell type-specific modes of action; a suggestion reinforced by the contrasting observations that Tnmd causes proliferation of tendon-derived cells but inhibits proliferation of vascular-derived cells.^9^ Our results are in line with the study of Jiang *et al.*^14^ as we showed *Tnmd*^*−/−*^ scars had significantly higher adipocyte accumulation and also that *Tnmd*^*−/−*^ TSPCs had a higher rate of differentiation into adipocytes. We propose that the different regulatory mechanisms of the Tnmd signaling pathway are involved in different cell types, which will be revealed when the *Tnmd* molecular network is finally mapped.

*Pparγ* is a transcriptional master regulator of adipogenic differentiation and stimulates adipogenesis.^53, 54, 55, 56^ Here, we showed that concomitantly with the higher adipocyte numbers, the lack of *Tnmd* significantly enhanced the expression levels of *Pparγ* and *Lpl in vivo and in vitro*. Furthermore, we observed that the absence of *Tnmd* results in dysregulated expression of key tendon transcription factors and ECM genes and proteins, which in turn may lead to altered scar composition and thereby increased lipid accumulation. Gehwolf *et al.*^57^ revealed that loss of the expression of the ECM protein Sparc drives adipocyte differentiation in tendons. Our study does not differentiate whether the pathways underlying the induction of adipogenesis either by *Pparγ* upregulation or by changes in ECM properties operate independently of each other or in an interdependent manner.^57, 58^ We can not provide a conclusive answer to this question, but we propose that *Tnmd* may strongly influence adipogenesis during tendon healing through the regulation of *Pparγ* expression, ECM composition and/or by preventing TSPC adipocyte commitment.

In summary, we created an Achilles tendon injury model in *Tnmd*^*−/−*^ mice, that showed that loss of *Tnmd* results in inferior tendon repair characterized by increased adipocyte accumulation, reduced cell density, proliferation and CD146-positive cells, increased apoptotic and p53-expressing cells, M1:M2 macrophage ratio changes, abnormal expression of tendon-related genes, and augmented fibrovascular scar composition. Concomitant *in vitro* analysis of *Tnmd*^*−/−*^ TSPCs revealed significantly reduced migratory and proliferative capacities, but upregulated adipogenic gene marker levels and accelerated differentiation down this lineage. Thus, our results suggest that Tnmd is required for prevention of adipocyte accumulation and fibrovascular scar formation during the early phase of tendon healing.

## Materials and Methods

### Animal model and surgical procedure

*Tnmd*^*−/−*^ mice and their WT littermates were used in this study. The generation of the *Tnmd*^*−/−*^ mice and their primary phenotype were described by Docheva and co-workers.^11, 12, 13^ All the mice were on a C57BL/6 J background. Surgical procedures were performed as previously described by Palmes *et al.*^15^ with 6-month old mice that had reached skeletal maturity. In brief, (1) after anesthesia, the skin above the left Achilles tendon was opened from the gastrocnemius muscle to the calcaneus; (2) using sterile scissors, the tendon proper (~5 mm above the calcaneus) of the Achilles tendon was fully resected; (3) the tendon ends were then connected with modified Kirchmayr 8-0 Dermalon suture and further supported with single 10-0 Dermalon circular suture; (4) in order to avoid suture failure due to overstretching of the operated tendons, the movement of the talocrural joint was restricted by a cerclage that was inserted through the tibiofibular fork and fixed between the calcaneus and the plantar aponeurosis. This assured a more limited degree of talocrural joint extension (~30%) but still allowed a tensile load to be actively transferred to the healing Achilles tendon; and (5) the skin was closed. The tendons were given eight days for repair, corresponding to the early phase of tendon healing, after which the animals were euthanized and the whole hind limb including the Achilles tendon-gastrocnemius muscle-calcaneus complexes were dissected and histologically processed as described below. All procedures for animal handling prior, during and after surgery were approved by the Animal Care and Use Committee of the Bavarian Government (Grant Nr. 55.2-1-54-2531-57-08). Bio-statistical design of the group size was based on the default values of *α*=0.05 and *β*=0.8 for type one error and for the power as well as on pilot histological data for each genotype, resulting in eight animals per group.

### Histomorphometry

Achilles tendons within the hind limbs were fixed in 4% paraformaldehyde (PFA; Merck, Darmstadt, Germany) overnight at 4 °C. After fixation, specimens were decalcified in 10% ethylene diamine tetraacetic acid (EDTA)/phosphate buffered saline (PBS) pH 8.0 (Sigma-Aldrich, Munich, Germany) for 7 days, and embedded in paraffin or cryogenic media and then sectioned at 5 or 10 *μ*m for paraffin and frozen specimens, respectively. Every 10th slide was stained with HE and slides with comparable regional planes between genotypes (where the whole complex of gastrocnemius muscle-Achilles tendon-calcaneus was exposed) were selected for in-depth investigation. To analyze the total histological scores on HE-stained slides we used the established histological scoring system of Stoll *et al.*^17^ given in Supplementary Table 1. To reveal the ectopic endochondral ossification in the scar tissue, safranin O staining was applied using the standard histological protocol.

For immunofluorescence, the tissue sections were treated with 2 mg/ml hyaluronidase (Sigma-Aldrich, Steinheim, Germany) for 30 min at 37 °C in order to increase antibody permeability. After washing and blocking with 2% bovine serum albumin (BSA)/PBS (Sigma-Aldrich), primary antibodies against biglycan, CD68, CD80, CD146, CD163, collagen I, collagen III, collagen IV, Comp, decorin, elastin, fibromodulin, fibronectin, F4/80, lumican, perilipin, p53 and Tnmd (all from Abcam, Cambridge, UK; except for Tnmd, which was provided by Denitsa Docheva) were applied overnight at 4 °C. Next day, corresponding Alexa Fluor 546-labeled secondary antibodies (all from Life technology, Carlsbad, CA, USA) were applied for 1 h. Then, sections were counter-stained with 4',6-diamidino-2-phenylindole (DAPI) (Life Technology) and mounted with fluoroshield (Sigma-Aldrich). To detect proliferating cells, 90 min prior to euthanasia all mice received intraperitoneal injection with BrdU (50 *μ*g/g body weight). BrdU detection was performed with a BrdU kit according to the manufacturer’s instructions (Roche Applied Science, Penzberg, Germany). To analyze apoptotic cells numbers, TUNEL assay was performed according to the manufacturer’s instructions (Abcam). Photo-micrographs were taken on the Observer Z1 microscope equipped with the Axiocam MRm camera (Carl Zeiss, Jena, Germany). In general, all histomorphometry experiments, unless specified otherwise in the text, were reproduced in with 8 animals per group and representative images are shown.

In order to analyze biglycan, Comp and fibronectin levels, an automated quantitative image analysis was performed as described in the literature.^18^ In brief, using ImageJ (National Institutes of Health, Bethesda, MD, USA), the following algorithm was applied: (1) area of interest was manually designated using the ‘drawing/selection’ tool; (2) ‘set measurements for area, integrated density and mean gray value’ was selected from the analyze menu; (3) the corrected total cryosection fluorescence (CTCF) representing the biglycan, Comp and fibronectin expression detected were calculated as follows CTCF=media of integrated density−(media of area of selected area × mean fluorescence). Three animals per group were analyzed.

Scar nuclear density was determined on DAPI staining with ImageJ according to Hsieh and co-workers.^18^ All cell nuclei (DAPI) in 3 images per scar from 3 sections per animal with 8 animals per group were counted. To analyze adipocytes (perilipin), blood vessels (collagen IV), cell proliferation (BrdU), apoptotic cells (TUNEL and p53), TSPC/MSC cells (CD146) and macrophages (CD68, CD80, CD163, and F4/80) quantification of labeled cell per scar tissue was carried out for each staining on 8 animals per group. Each animal was represented with 3 different tissue sections with comparable planes between genotypes. The results were averaged per animal and shown as final mean and standard deviation (S.D.) between the 8 animals per group. The above information is given in the figure legends (8 animals per group; each animal represented by 3 tissue sections).

### Mouse TSPCs isolation and cell culture

Mouse TSPCs were isolated as previously described by Alberton and co-workers^13^ from tendons of two uninjured *Tnmd*^*−/−*^ and WT 6-month-old mice. Tendon tissues were enzymatically treated overnight with collagenase II (Worthington, Lakewood, NJ, USA) in Dulbecco’s modified Eagle’s medium (DMEM)/Ham’s F-12 (1:1) (Biochrom, Berlin, Germany) supplemented with 10% fetal bovine serum (FBS), 1% l-ascorbic-acid-2-phosphate (both from Sigma-Aldrich, Steinheim, Germany), 1% minimum essential medium (MEM)-Amino Acid and 1% penicillin/streptomycine (Pen/Strep) (both from Biochrom, Berlin, Germany). Then, the cell suspension was filtered through 70 *μ*m nylon mesh, centrifuged at 500 × *g* for 5 min, and resuspended in fresh culture media. TSPCs were grown at 37 °C and 5% CO_2_ and passaged when 70% confluent with the culture media changed every third day. Cells in passages 1–3 were used for experiments.

### *In vitro* wound healing assay

These experiments were carried out according to our previously published protocol.^59^ Shortly, 1 × 10^4^ cells per cm^2^ were plated on collagen I- (20 *μ*g/Ml; Millipore, Billerica, MA, USA) or fibronectin-coated (10 *μ*g/Ml; Sigma-Aldrich, Steinheim, Germany) 6-well plates in low serum (2%) medium and were allowed to form confluent cell layers for 48 h. Prior to imaging, the layers were scratched multiple times. Time lapse photography was performed at 4 frames per h for 24 h. For each group, the areas of 12 scratches were measured at 9 different time points from 4 independent experiments using the ImageJ ‘wound healing’ tool.

### Migration analysis

Migration analysis was performed similarly to our previous studies.^59, 60^ For random migration, 1.5 × 10^3^ cells/cm^2^ of *Tnmd*^*−/−*^ and WT TSPCs were seeded on collagen I- (20 *μ*g/ml) or fibronectin-coated (10 *μ*g/ml) 6-well plates and incubated for 2 h before imaging. Time lapse photography was performed at 4 frames per h for 48 h. The image data was extracted with AxioVisionLE software (Carl Zeiss, Jena, Germany) and individual cell tracks were analyzed with ImageJ. Random migration was expressed by calculating the forward migration index (FMI; the ratio of the vector length to the migratory starting point), velocity, and accumulated (cumulative track length) and Euclidian (the ordinary straight-line length between two points) distances. Results of random TSPCs migration measurements consist of 3 independent time lapse movies of two *Tnmd*^*−/−*^ and WT TSPC donors as a total number of 70–80 TSPC per genotype were tracked.

### CyQUANT assays

1.5 × 10^3^ cells per well were plated in 6-well plates, and the CyQUANT assay detection was performed according to the manufacturer’s instructions (Invitrogen, Eugene, OR, USA) after 0, 4, 8 and 12 days of cell culture, respectively. CyQUANT assay was repeated independently in 4 experiments per time point with two TSPC donors/genotypes.

### Adipogenic differentiation assays

These experiments were carried out according to our previously published protocol.^13^ Briefly, 8 × 10^3^ cells/cm^2^ TSPCs were seeded in triplicates in 6-well plates, and were cultivated in an induction media for 5 days (DMEM-high glucose with 10% FBS, 1 *μ*M dexamethasone, 200 *μ*M indomethacin, 0.01 mg/ml insulin, and 500 *μ*M 3-isobutyl-1-methylxanthine; all from Sigma-Aldrich, Steinheim, Germany) followed by 2 days in preservation media (DMEM-high glucose medium supplemented with 10% FBS, 0.01 mg/ml insulin). The process was repeated for 21 days. The adipogenic differentiation was estimated by BODIPY 493/503 staining of neutral lipid droplets (Thermo Fisher Scientific, Waltham, MA, USA) and AdipoRed assay (Lonza, Walkersville, MA, USA). Staining was carried out according to the manufacturer’s instructions. Using the automatic color pixel quantification tool in the Adobe Photoshop CS5 software, the BODIPY 493/503 staining-positive areas were estimated and calculated as a percentage of the image total pixel size. Using a fluorimeter (Tecan, Männedorf, Switzerland), AdipoRed assays were measured with excitation at 485 nm and emission at 572 nm. BODIPY 493/503 staining and AdipoRed assay were repeated in 4 independent experiments.

### ELISA

The protein levels of biglycan and fibronectin were analyzed by ELISA. TSPCs (8 × 10^3^ cells/cm^2^) were seeded in 6-well plates. After 3 days, the cell supernatant and cell RIPA protein lysates were collected and frozen. Before ELISA, the total protein of all samples was measured via DC protein assays (BioRad, Munich, Germany). Secreted biglycan and fibronectin were determined using mouse biglycan and fibronectin ELISA kits (Cloud-Clone Corp, Katy, TX, USA; and Aviva Systems Biology, San Diego, CA, USA; respectively) according to the manufacturer’s instructions. Two independent ELISA measurements were done with two donors/genotypes. The data was expressed as target-specific concentration to total protein content.

### Semi-quantitative and qRT-PCR

Total RNA from tendon tissue and adipogenic-stimulated TSPCs was isolated with Qiagen RNeasy Mini kit (Qiagen, Hilden, Germany) and used for semi-quantitative and qRT-PCR. For cDNA synthesis, 1 *μ*g total RNA and AMV First-Strand cDNA Synthesis Kit (Invitrogen) were used. Semi-quantitative PCR was performed with Taq DNA Polymerase (Invitrogen) in MGResearch instrument (BioRad, Munich, Germany). For Primer sequences and PCR conditions: *Pparγ* forward 5′ctccgtgatggaagaccactc3′, reverse 5′agactcggaactcaatggc3′ *Lpl* forward 5′gtctggctgacactggacaa3′, reverse 5′tgggccattagattcctcac3′ *Fabp4* forward 5′gaagcttgtctccagtcaaaa3′, reverse 5′agtcacgcctttcataacacat3′ *Gapdh* forward 5′gagaggccctatcccaactc3′, reverse 5′gtgggtgcagcgaactttat3′ PCR was performed with incubation at 94 °C for 5 min followed by 30 cycles of a three step temperature program of 1 min at 94 °C, 20 s at 60 °C, and 30 s at 72 °C. The PCR reaction was terminated after a 7 min extension at 70 °C. The band intensity of the amplified products in the gel was visualized, photographed and analyzed using a gel imager (Vilber Lourmat, Eberhardzell, Germany). The relative gene expression was quantified by densitometry and normalized to the amount of Gapdh with ImageJ and presented as fold-change to WT. Quantitative RT-PCR of adipogenic and tenogenic associated gene markers was performed using RealTime Ready Custom Panel 96–32+ plates (https://configurator.realtimeready.roche.com) according to the manufacturer’s instructions (Roche Applied Science, Mannheim, Germany). Briefly, PCR reactions were pipetted on ice and each well contained 10 *μ*l LightCycler 480 probes master mix, 0.2 *μ*l cDNA and 9.8 *μ*l PCR grade water. Plates were subsequently sealed and centrifuged down for 15 s at 2100 rpm. The relative gene expression was calculated as describe by Dex and co-workers.^12^ All PCR results have been reproduced independently in five experiments.

### Statistical analysis

Statistical differences between two groups were determined using two-tailed unpaired Student’s *t*-test, or two-tailed non-parametric Mann–Whitney test. Sample size and experimental reproduction are indicated for each method. Results are presented as mean±S.D. Differences were considered statistically significant according to values of *P<0.05, **P<0.01 and ***P<0.001.
