# Supplementary material for: Tenomodulin is essential for prevention of adipocyte accumulation and fibrovascular scar formation during early tendon healing
Source: Cell Death Dis. 2017 Oct 12;8(10):e3116–. doi: 10.1038/cddis.2017.510 (PMC5682675; doi:10.1038/cddis.2017.510)
Supplement: Supplementary Information [file cddis2017510x1.docx]

**Supplementary Information**

**Supplementary Figure legends**

**Supplementary Figure 1. (a)** No profound differences in the fluorescent signal intensities were observed for collagen I and III, decorin, elastin, fibromodulin and lumican in the healing region in both *Tnmd^-/-^* and WT mice at 8 days postoperatively. **(b)** Picrosirious red-stained sections (where larger collagen fibers are bright yellow or orange, and thinner ones, including reticular fibers, are green) were analyzed via polarized light microscopy showing that the scar-like tissue and tendon ends in *Tnmd^-/-^* mice contained more thicker collagen fibers than WT mice (8 animals per group; each animal represented by 3 tissue sections). The polarized light analyzer/polarizer parameters were fixed between samples permitting direct cross-comparison and were not varied for optimal imaging. S, scar; T, tendon. Scale bars: 50 μm

**Supplementary Figure 1**


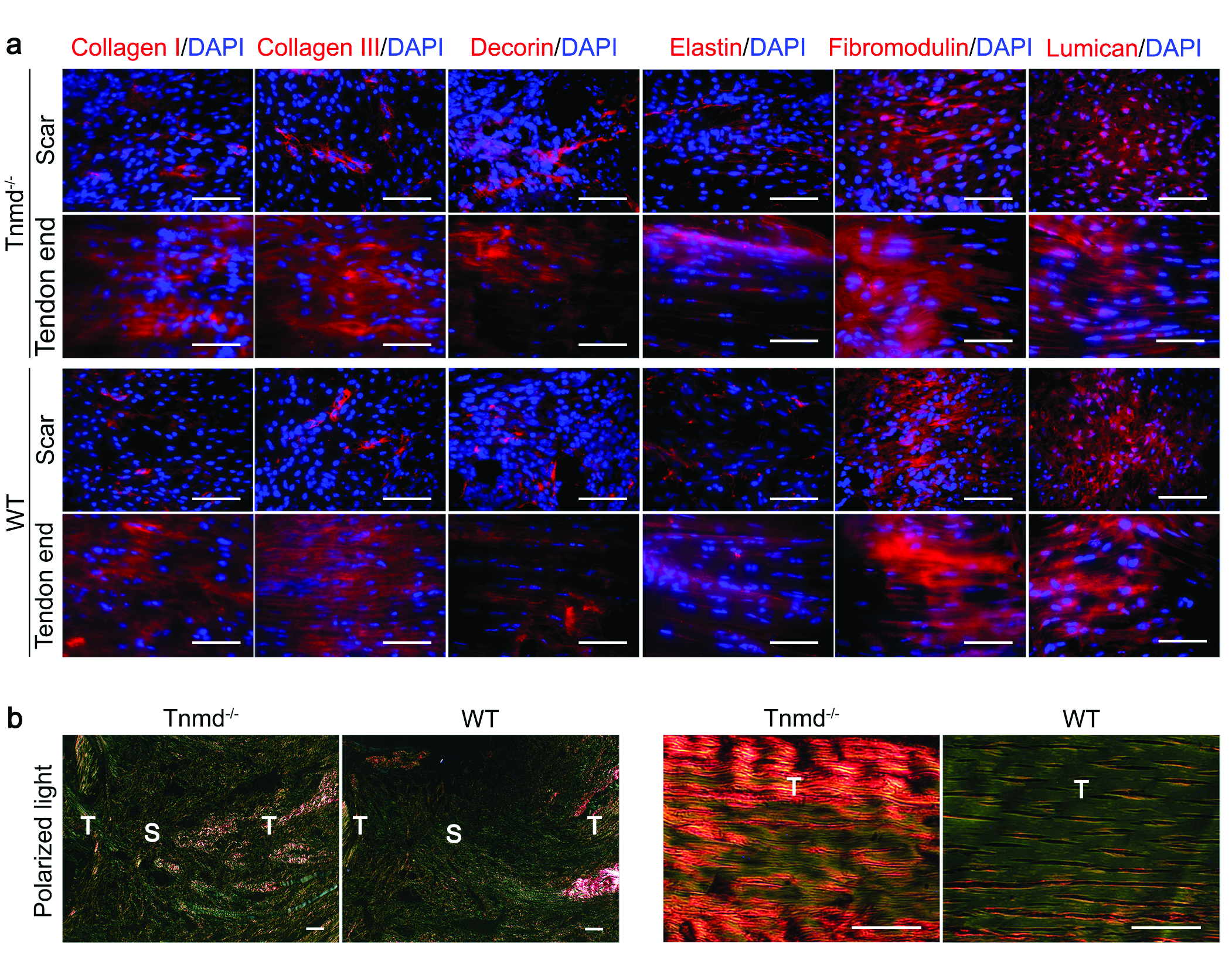


**Supplementary Table S1** Histological scoring system^1^

| Evaluated parameters | Points |
| --- | --- |
| *Extracellular matrix (ECM) organization of the whole tendon*  Wavy, compact and parallel arranged collagen fibers  In part compact, in part loose or not orderly  Loosely composed, not orderly (“granulation” tissue) | 2  1  0 |
| *Proteoglycan content (Safranin Orange staining)*  Normal  Focally increased | 1  0 |
| *Cellularity/cell-matrix-ratio*  Physiological  Locally increased cell density  Increased cell density or decreased ECM content | 2  1  0 |
| *Cell alignment*  Uniaxial  Areas of irregularly arranged cells (10-50%)  More than 50% of cells with no uniaxial alignment | 2  1  0 |
| *Cell distribution*  Homogeneous, physiological  Focal areas of elevated cell density (cell clustering) | 1  0 |
| *Cell nucleus morphology*  Predominantly elongated, heterochromatic cell nuclei (tenocytes)  10-30% of the cells possess large, oval, euchromatic or polymorph heterochromatic nuclei  Predominantly larger, oval, euchromatic or polymorph, heterochromatic nuclei | 2  1  0 |
| *Organization of repair tissue of the tendon callus*  Homogeneous (whole tissue with similar composition)  Locally heterogeneous tissue composition  Whole tissue composition completely changed | 2  1  0 |
| *Transition from defect to normal tissue*  Scaffold integrated, no gaps at the margin visible  Recognizable transition  Abrupt transition, splitting/gaps detectable, callus tissue | 2  1  0 |
| *Configuration of callus*  Normal, only in the defect area, locally confined  Strong, change of whole tendon, thickened | 1  0 |
| *Degenerative changes/tissue metaplasia*  Non existing  Moderate formation of oedema  Intense oedema with inclusion of fat, cell and/or fibers destruction, fibrin deposition, gaps  Assembly of cartilage or bone (Safranin Orange staining) | 3  2  1  0 |
| *Vascularization in the defect area*  Hypo-vascularized, like surrounding tendon (small capillaries)  Hyper-vascularized (increased numbers of small or larger capillaries) | 1  0 |
| *Inflammation*  No inflammatory cell infiltrates  Infiltrating inflammatory cell types (neutrophils, macrophages, foreign-body/giant cell) | 1  0 |

**Supplementary Materials and Methods**

**Polarized light microscopy.** The collagen fiber orientation was assessed in picrosirius red-stained sections (where larger collagen fibers are bright yellow or orange, and thinner ones, including reticular fibers, are green), as were described by [Hsieh](https://www.ncbi.nlm.nih.gov/pubmed/?term=Hsieh%20CF%5BAuthor%5D&cauthor=true&cauthor_uid=27763655) *et al*.^2^ and Tokunaga and co-workers.^3^ Microscopy was performed with 10× objective supplemented with a polarized light filer mounted on Axioskope 2 microscope (Carl Zeiss, Jena, Germany). For optimal imaging the transmission axis of the analyzer was with an angle of 54º to the axis of the polarizer in the analysis of all samples allowing direct cross-comparison between the genotypes.

**References**

1. [Stoll C](https://www.ncbi.nlm.nih.gov/pubmed/?term=Stoll%20C%5BAuthor%5D&cauthor=true&cauthor_uid=21474176), [John T](https://www.ncbi.nlm.nih.gov/pubmed/?term=John%20T%5BAuthor%5D&cauthor=true&cauthor_uid=21474176), [Conrad C](https://www.ncbi.nlm.nih.gov/pubmed/?term=Conrad%20C%5BAuthor%5D&cauthor=true&cauthor_uid=21474176), [Lohan A](https://www.ncbi.nlm.nih.gov/pubmed/?term=Lohan%20A%5BAuthor%5D&cauthor=true&cauthor_uid=21474176), [Hondke S](https://www.ncbi.nlm.nih.gov/pubmed/?term=Hondke%20S%5BAuthor%5D&cauthor=true&cauthor_uid=21474176), [Ertel W](https://www.ncbi.nlm.nih.gov/pubmed/?term=Ertel%20W%5BAuthor%5D&cauthor=true&cauthor_uid=21474176) *et al*. Healing parameters in a rabbit partial tendon defect following tenocyte/biomaterial implantation. [*Biomaterials*](https://www.ncbi.nlm.nih.gov/pubmed/?term=Healing+parameters+in+a+rabbit+partial+tendon+defect+following+tenocyte%2Fbiomaterial+implantation) 2011; **32**: 4806-4815.
2. [Hsieh CF](https://www.ncbi.nlm.nih.gov/pubmed/?term=Hsieh%20CF%5BAuthor%5D&cauthor=true&cauthor_uid=27763655), [Alberton P](https://www.ncbi.nlm.nih.gov/pubmed/?term=Alberton%20P%5BAuthor%5D&cauthor=true&cauthor_uid=27763655), [Loffredo-Verde E](https://www.ncbi.nlm.nih.gov/pubmed/?term=Loffredo-Verde%20E%5BAuthor%5D&cauthor=true&cauthor_uid=27763655), [Volkmer E](https://www.ncbi.nlm.nih.gov/pubmed/?term=Volkmer%20E%5BAuthor%5D&cauthor=true&cauthor_uid=27763655), [Pietschmann M](https://www.ncbi.nlm.nih.gov/pubmed/?term=Pietschmann%20M%5BAuthor%5D&cauthor=true&cauthor_uid=27763655), [Müller PE](https://www.ncbi.nlm.nih.gov/pubmed/?term=M%C3%BCller%20PE%5BAuthor%5D&cauthor=true&cauthor_uid=27763655) *et al*. Periodontal ligament cells as alternative source for cell-based therapy of tendon injuries: in vivo study of full-size Achilles tendon defect in a rat model. [*Eur Cell Mater*](https://www.ncbi.nlm.nih.gov/pubmed/?term=Periodontal+ligament+cells+as+alternative+source+for+cell-based+therapy+of+tendon+injuries%3A+in+vivo+study+of+full-size+Achilles+tendon+defect+in+a+rat+model) 2016; **32**: 228-240.
3. [Tokunaga T](https://www.ncbi.nlm.nih.gov/pubmed/?term=Tokunaga%20T%5BAuthor%5D&cauthor=true&cauthor_uid=26311443), [Shukunami C](https://www.ncbi.nlm.nih.gov/pubmed/?term=Shukunami%20C%5BAuthor%5D&cauthor=true&cauthor_uid=26311443), [Okamoto N](https://www.ncbi.nlm.nih.gov/pubmed/?term=Okamoto%20N%5BAuthor%5D&cauthor=true&cauthor_uid=26311443), [Taniwaki T](https://www.ncbi.nlm.nih.gov/pubmed/?term=Taniwaki%20T%5BAuthor%5D&cauthor=true&cauthor_uid=26311443), [Oka K](https://www.ncbi.nlm.nih.gov/pubmed/?term=Oka%20K%5BAuthor%5D&cauthor=true&cauthor_uid=26311443), [Sakamoto H](https://www.ncbi.nlm.nih.gov/pubmed/?term=Sakamoto%20H%5BAuthor%5D&cauthor=true&cauthor_uid=26311443) *et al*. FGF-2 stimulates the growth of tenogenic progenitor cells to facilitate the generation of Tenomodulin-positive tenocytes in a rat rotator cuff healing model. [*Am J Sports Med*](https://www.ncbi.nlm.nih.gov/pubmed/?term=FGF-2+Stimulates+the+Growth+of+Tenogenic+Progenitor+Cells+to+Facilitate+the+Generation+of+Tenomodulin-Positive+Tenocytes+in+a+Rat+Rotator+Cuff+Healing+Model) 2015; **43**: 2411-2422.
